# Trichina

**Published:** 1872-04

**Authors:** 


### TRICHINA.

There is, perhaps, no stronger and longer race of men in the world than the Tennesseeans and Kentuckians born within the century. The editor knows whole families in Kentucky who were many of them over seven feet high; the daughters were six feet. Still it may be said, without exaggeration, that the meat of hogs is on every farmer's table each day in the year, eaten probably on an average at two meals out of the three. And yet many Germans die of the most painful of all maladies from eating pork; the difference being that the Western people require pork in all its forms to be particularly well cooked — cooked most thoroughly; the Germans, from habit, eat it raw. Charles Martin's wife and two children, at Cleveland, Ohio, ate freely of raw, smoked sausages. Within three days the father and mother died with horrible sufferings; the children lingered, but may recover. A brother had a portion of the same animal well cooked and placed on his table; it was eaten without fear, and with no ill result. The practical point is too plain not to be noted.

